# Malic enzyme 3 mediated the effects of malic acid on intestinal redox status and feed efficiency in broilers

**DOI:** 10.1186/s40104-025-01163-3

**Published:** 2025-02-24

**Authors:** Hongfeng Jiang, Genghui Li, Xue Yang, Xiaohua Feng, Penglin Li, Huisi Yang, Demin Cai, Qingyan Jiang, Gang Shu

**Affiliations:** 1 State Key Laboratory of Swine and Poultry Breeding Industry, Guangdong Province Key Laboratory of Animal Nutritional Regulation, College of Animal Science, South China Agricultural University, Guangzhou, Guangdong 510642 China; 2 College of Animal Science and Technology, Yangzhou University, Yangzhou, China; 3 United Bio-Tech Co., Ltd., Guangzhou, China

**Keywords:** Colitis, Feed efficiency, Malic acid, Malic enzyme 3, Redox

## Abstract

**Background:**

Intestinal oxidative stress serves as an endogenous host defense against the gut microbiota by increasing energy expenditure and therefore decreasing feed efficiency (FE). Several systems coordinately regulate redox balance, including the mitochondrial respiratory chain, nicotinamide adenine dinucleotide phosphate (NADPH) oxidase, and different antioxidants. However, it remains unclear which redox balance compartments in the intestine are crucial for determining FE.

**Results:**

In this study, we first screened the key targets of different metabolites and redox balance-related gene expression in broiler ceca. We then constructed a mouse colitis model to explore malic acid (MA) ability to alleviate intestinal inflammation. We further used controlled release technology to coat MA and investigated its effects on the intestinal redox status and FE in vivo. Finally, we examined the underlying mechanism by which MA modulated redox status using a porcine intestinal epithelial cell jejunum 2 (IPEC-J2) cell model in vitro. Our results demonstrated that the MA/malic enzyme 3 (*ME3*) pathway may play an important role in reducing oxidative stress in the broiler cecum. In addition, colon infusion of MA attenuated inflammatory phenotypes in the dextran sulfate sodium salt (DSS) induced mouse colitis model. Then, dietary supplementation with controlled-release MA pellet (MAP) reduced the feed to gain (F/G) ratio and promoted chicken growth, with reduced oxidative stress and increased bacterial diversity. Finally, the in vitro IPEC-J2 cell model revealed that ME3 mediated the effect of MA on cellular oxidative stress.

**Conclusion:**

In summary, our study firstly revealed the important role of the MA/*ME3* system in the hindgut of broiler chickens for improving intestinal health and FE, which may also be crucial for the implications of colon inflammation associated diseases.

**Supplementary Information:**

The online version contains supplementary material available at 10.1186/s40104-025-01163-3.

## Introduction

In large-scale breeding, feed costs constitute approximately 60% of the total cost of livestock and poultry breeding [1]. Therefore, improving feed efficiency (FE) is crucial for increasing the efficiency of animal husbandry. Oxidative stress emerges as the primary factor reducing FE in broilers, manifesting in decreased feed intake, impaired growth performance, and immunosuppression. This stress is indicative of diminished activity within the mitochondrial respiratory chain complex [2–4]. Reactive oxygen species (ROS) disrupt redox homeostasis and cause severe damage to the host. There are effective protective systems against the potential deleterious effects of oxidative damage, including enzymatic and non-enzymatic antioxidants [5]. Enzymatic antioxidants include mainly glutathione redox and the thioredoxin/peroxiredoxin system, and non-enzymatic antioxidants include vitamin C, vitamin E, and so on [6].

The gastrointestinal tract serves not only as the site for feed digestion and absorption, but also the primary source of ROS generation [7]. Researches have demonstrated that hydrogen peroxide (H_2_O_2_) is produced through various mechanisms, including nicotinamide adenine dinucleotide phosphate (NADPH) oxidase, the mitochondrial electron transport chain, and D-amino acid oxidase etc., which are important for maintaining the intestinal microbiota balance and preventing microbiota disorders [8–10]. However, excess H_2_O_2_ can also cause oxidative damage to the mucosa, leading to reduced growth performance and increased inflammation [11, 12]. In our previous study, we found that oxidative stress was more pronounced in the hindgut compared to the foregut [13], which was consistent with the findings from other publications [14]. Therefore, delineating the origin of ROS and mitigating oxidative stress in the hindgut might be important strategies for enhancing animal growth performance and intestinal health.

In this study, we first screened the key targets of different metabolites and redox balance-related gene expression in the cecum of broilers. The mouse model of colitis was subsequently used to explore the role of malic acid (MA) in alleviating intestinal inflammation. We further used controlled-release technology to coat MA pellets and investigated their effects on the intestinal redox status and FE in vivo. Subsequently, the underlying mechanism of MA regulation of redox status was investigated in vitro by the porcine intestinal epithelial cell jejunum 2 (IPEC-J2) cell model. This study establishes a vital role for developing new nutritional strategies to improve the chicken FE and offers insights into the treatment of colon inflammation-associated diseases.

## Materials and methods

All procedures for animal care were approved by the South China Agricultural University Animal Care and Use Committee (Guangzhou, China; Project number SYXK 20220136).

### Animals, housing, experimental design and diets

To find key regulatory targets in chickens with different growth performance, in trial 1, 30 21-day-old Arbor Acre (AA) male × Ross female cross broilers (Qingnong Technology Co., Ltd., Guangdong, China, initial body weight 377 ± 8.87 g) were housed at the Zengcheng Teaching and Research Base (South China Agricultural University, Guangdong, China) and allowed to drink (automatic device) and feed (daily manual addition) freely. The birds were not divided into groups and received only basal diets. Each bird was housed in a separate cage (25 cm × 30 cm × 30 cm) to avoid fecal disturbances, with controlled temperature/humidity controlled at (26 ± 1 °C/60% ± 10%) and 24 h light. The experiment lasted 24 d (3 d pre-feeding period and a 21-d formal period). All birds were slaughtered without fasting. The entire cecum on the same side of each chicken was sampled, including cecal contents and mucosa, and stored at −80 °C until analysis. Body weight was measured at 24 and 45 d, and feed intake was recorded weekly. Body weight gain (BWG), cumulative feed intake (FI) and feed-to-gain ratio (F/G) were calculated. In order to better understand the correlation between production performance and various indicators, we divided 30 chickens into 2 groups based on the 3 indicators of F/G, BWG, and FI. In particular, the average F/G of the 30 chickens was calculated to be 2.3, and we designated 15 chickens with a value higher than 2.3 as the high F/G group and 15 chickens with a value lower than 2.3 as the low F/G group. Similarly, the mean BWG of the 30 chickens was calculated to be 650 g. We designated the 15 chickens with values higher than 650 g as the high BWG group and the 15 chickens with values lower than 650 g as the low BWG group; the mean FI of the 30 chickens was calculated to be 1,700 g. We designated the 15 chickens with values higher than 1,700 g as the high FI group and the 15 chickens with values lower than 1,700 g as the low FI group.

We wondered if MA could alleviate hindgut inflammation and offers insights into the treatment of colon inflammation-associated diseases. In trial 2, 32 male 8-week-old C56BL/6 mice (acclimated for 1 week and initial body weight 19.03 ± 0.09 g, Zhuhai Baishitong Biotechnology Co., Ltd., Zhuhai, China,) were housed in a temperature/humidity-controlled facility (23 ± 3 °C/70% ± 10%) under a 12 h light–dark cycle. Unless otherwise stated, the mice were fed regular mouse chow (18.0% protein, 4.5% fat and 58% carbohydrate; Guangdong Medical Science Experiment Center, Guangdong, China) and water throughout the experiment. For colon perfusion, a 1-mL syringe barrel fitted with a flexible gavage needle (50 mm) was used. The mouse was grasped from the back with the abdomen facing upward and the head tilted downward at 45°. The abdomen of the mouse was gently rubbed so that the feces could be discharged, after which the tube was inserted through the anus, and after reaching the colon the liquid was instilled according to the body weight, and then the tube was withdrawn. Keep this position for 30 s to allow all the fluid instilled into the intestinal tract. The 32 mice were divided into 4 groups, with 8 replicates per group (in the same 2 cages). The trial lasted 21 d. Perfusions were administered every 3 d as follows: 1) Control group: Perfused with saline (0.9% sodium chloride solution, pH = 7) and given free access to water for 21 d. 2) MA group: Perfused with malic acid (MA) (10 μg/g, pH = 7; Sangon Biotech, Shanghai, China) and given free access to water for 21 d. 3) DSS group: Perfused with saline for 21 d, with 3% dextran sulfate sodium salt (DSS) added to drinking water for the last 6 d. 4) DSS + MA group: Perfused with MA for 21 d, with 3% DSS added to drinking water for the last 6 d. All the mice were culled at the end of the trial. Colons and livers from all mice were collected and colons stored at −80 °C until analysis.

In order to be applied in production practice and target the hindgut, we further used controlled-release technology to coat MA pellets and investigated their effects on the intestinal redox status and FE in vivo. In trial 3, 120 19-day-old Arbor Acre male × Ross female cross broilers (Qingnong Technology Co., Ltd., Guangdong, China, initial body weight 283.12 ± 2.46 g) were housed as the same with trial 1. Birds were randomly assigned to 4 groups, each group had 30 replicates with 1 bird per replicate. The groups were as follows: control group (Con), blank pellet group (containing 1‰ blank pellets, negative control, NC), MA group (containing 1‰ MA, pH = 7, MA), and the coated MA pellet group (containing 1‰ MA, MAP). Pellets detailed in the following paragraphs. Growth performance was recorded and calculated as in trial 1. This trial lasted 3 weeks. At the end of this experiment, 15 chickens were randomly selected from each group for sampling. In trial 3, slaughter weight, liver weight, colon length, breast muscle weight, thigh muscle weight, and abdominal fat weight were recorded. Serum, cecal contents and mucosa (one side) were collected and stored at −80 °C until analysis.

The composition and nutrient levels of the basal diets are shown in the supplementary materials (Tables S1). Huayang Feedstuff Co., Ltd. (Foshan, China) provided the diet for broilers.

### Cell culture and treatment

IPEC-J2 cells were donated by Academician Yin Yulong from the College of Animal Science, South China Agricultural University. The cells were cultured in Dulbecco's modified Eagle medium (DMEM) supplemented with 10% fetal bovine serum, 100 U/mL penicillin, and 100 μg/mL streptomycin in a 5% CO_2_ incubator at 37 °C. When IPEC-J2 cells reached 50% confluency, we replaced fresh DMEM containing 0 mmol/L, 0.1 mmol/L, or 1 mmol/L MA (dissolved in PBS, pH = 7) and cultured them for 24 h. Afterward, the cells were washed 3 times with pre-cooled PBS, and the cell plates were stored at −80 °C for subsequent experiments.

### Pellet preparation

The pellets were prepared by College of Pharmacy, Anhui University of Chinese Medicine using an extrusion and spheronization technique [15]. Briefly, 500 g of MA was dissolved in 700 mL water and adjusted to pH = 7 using NaOH. Microcrystalline cellulose (Henan Wanbang Chemical Technology Co., Ltd., 50% mass of the total pellets) was added as an excipient for shaping. Blank pellets contained only microcrystalline cellulose. The pellets were rounded in a granulator (LBLX-360, Jiujiang Dongsheng Machinery Manufacturing Co., Ltd., China). The prepared pellets were then dried at a constant temperature of 55 °C for 12 h to ensure an optimum and uniform humidity level. Based on the material the chicken feeds [16], we collected 30–40 mesh pellets, and each pellet weighed approximately 0.03 mg. Acrylic resin (Youcare Pharmaceutical Group, Anhui, China) was coated on the pellets using a fluidized bed coater (LDP-1.5, Changzhou Jingtian Drying Granulation Equipment Co., Ltd., China).

### Release test in vitro

Release test was conducted to determine the profiles of pellet release. Gastrointestinal fluid included simulated gastric fluid (SGF) (R30386, Shanghai yuanye Biotechnology Co., Ltd., pH = 2, containing sodium chloride, dilute acid, pepsin, and so on), artificial small intestine fluid (SIF) (R30384, Shanghai yuanye Biotechnology Co., Ltd., pH = 6.8, including phosphates, pancreatic enzymes, and so on), and large intestine fluid (LIF) (pH = 7.5, adjusted pH with NaOH in SIF). The pellets and gastric fluid mixture were mixed at 33% (w/v) ratio. Release test lasted for 12 h at different pH values. For pellet releases, we optimized using a shaker (THZ-98AB, Bluepard) with reference to dissolution apparatus (708-DS, Agilent). All pellets were sequentially added to SGF (2 h), SIF (4 h) and LIF (4 h) in turn. Samples were taken periodically, centrifuged at 10,000 ×* g* for 5 min in an Eppendorf centrifuge (Eppendorf 5810R, Germany), and the supernatant was collected. Spectrophotometry (BIOTEK, Vermont, USA) was performed at a wavelength of 600 nm [17, 18].

### HPLC for organic acid analysis

High-performance liquid chromatography (HPLC, Model 1260, Agilent Technologies, Inc., USA) was used to analyze organic acid. The methods were performed as described previously [19]. Briefly, the contents/mucous were homogenized with methanol at a 1:9 ratio based on weight and centrifuged at 10,000 × *g* for 15 min at 4 °C. The supernatant was collected and filtered through a 0.22-μm syringe filter. Mixed standards (0, 7, 70, 135, 350, and 700 µg/mL) and a single standard (1 mg/mL) for each organic acid were used for chromatogram comparison. A Zorbax SB-Aq column (4.6 mm × 250 mm, 5 μm, Agilent) was used. The mobile phase for organic acids consisted of 25 mmol/L KH_2_PO_4_ (pH = 2.5) to separate acetic acid, MA, lactic acid, citrate, oxaloacetic acid, α-ketoglutarate (AKG) and succinic acid. Chromatograms were collected at 254 nm. A flow rate of 1 mL/min was used for the isocratic mode above. Aliquots of 20 µL were injected into the column, and determinations were performed at 25 °C.

### Histological staining and analysis

The mouse colon was dissected without squeezing, cut into approximately 1 cm pieces, and fixed in a 2-mL centrifuge tube containing 4% paraformaldehyde. The samples were then sent to Service Biotechnology Co. Ltd. (Wuhan, China) for paraformaldehyde fixation and paraffin embedding. Hematoxylin and eosin (H&E) staining was used for histological staining. The severity of colitis was assessed based on pathological and histological feature, as previously described [20]. Briefly, the scoring system was as follows: 0: No or mild inflammation with depletion of goblet cells in histological tissue sections (observed under an H600L microscope, Nikon, Japan). 1: Acute focal inflammation and crypt abscess formation. 2: Widespread inflammation with indications of thickening smooth muscle. 3: Ulcerations and cellular inflammation in tissue sections. 4: Signs of gangrene, necrosis, and mucosal tissue loss.

### Colon length and disease activity index

In trial 2, colon length was measured using a ruler. Clinical colitis severity was assessed by measuring body weight change, diarrhea, and bleeding as previously described [21]. Briefly, for diarrhea scores, 0 represented a firm and formed stool; 2 represented a soft and unformed stool; and 4 represented a liquid stool. We modified the bleeding scores: 0 represented normal stool; 2 represented bloody stool; and 4 represented bloody stool throughout the entire anus. Weight loss was scored as follows [22]: 0 for no significant weight loss, 1 for 1%–5% loss, 2 for 5%–10% loss, 3 for 10%–20% loss, and 4 for greater than 20% loss. The disease activity index (DAI) was the sum of weight loss score, diarrhea score, and bleeding score divided by 3.

### Immunofluorescence staining

Dihydroethidium (DHE) (D1008, UElandy) was used at a 1:1,000 dilution to stain frozen cecum sections (8 μm thick) [23]. TUNEL (C1086, Beyotime), Ki67(27309-1-AP, Proteintech), F4/80 (29414-AP, Proteintech), MUC2 (A14659, ABclonal), and LYZ (A0641, ABclonal) staining were performed on 5 μm paraffin slices. All the paraffin sections were dewaxed in water and underwent antigen retrieval using sodium citrate. Staining procedures were carried out according to the manufacturers' instructions.

Apoptotic cells were detected using a TUNEL Apoptosis Kit [24]. Paraffin sections were treated with proteinase K and labeled with terminal deoxynucleotide transferase (TdT) enzyme added along with a nucleotide mix containing fluorescein-dUTP conjugate, then washed with PBS. Proliferating cells were stained with Ki67, and macrophages with F4/80. Sections were blocked with 0.1% Tween in phosphate-buffered saline (PBST) containing 5% goat serum. Primary antibodies were diluted 1:1,000 in 5% goat serum PBST and incubated at 4 °C overnight. Secondary antibodies (goat anti-rabbit IgG H + L Cy3: JAC 111-165-045, 1:1,000, Jackson) were added to PBST and then incubated at room temperature for 1 h. Paneth cells were stained with LYZ and goblet cells with MUC2. Sections were blocked with Tris-buffered saline (TBS) containing 5% goat serum, washed with 0.1% Tween in TBS (TBST) and TBS, then incubated with primary antibodies (1:1,000 in TBS) at 4 °C overnight. Secondary antibodies (goat anti-rabbit IgG H + L Cy3: JAC 111-165-045, 1:1,000, Jackson) incubation was performed as described above, using TBS as the diluent. DAPI was used to stain nucleus. Finally, a fluorescence microscope (Nikon Instruments, Tokyo, Japan) was used for observation and imaging.

To assess cell proliferation, we used the Cell-Light EdU Apollo488 In Vitro Kit (C10310, RiboBio, Guangzhou, China). Cells were labeled with EdU and incubated for 12 h, then fixed with 4% paraformaldehyde. Apollo staining and Hoechst 33342 nuclear staining followed. An Annexin V-FITC/PI Apoptosis Assay Kit (40302ES50, Yeasen, Shanghai, China) was used for cell apoptosis evaluation. Annexin-V identifies early apoptotic cells, while PI marks late apoptotic and necrotic cells. We washed the cells with cooled PBS, then added binding buffer, Annexin V-FITC, and PI staining solution, after a 10-min incubation at room temperature in darkness. Hoechst 33342 was used to stain the cell nuclei.

### RNA interference

RNA interference was performed as previously described [25]. Malic enzyme 3 (ME3) siRNA and negative control (NC) siRNA were purchased from RiboBio (Guangzhou, China) and transfected into IPEC-J2 cells using Lipofectamine reagents (Invitrogen, Carlsbad, CA, USA) according to the manufacturer’s instructions. The sequences of the siRNA targeting ME3 were siME3-1 ((5′-CUCAACAAAUACCGCAACATT-3′ (sense) and 5′ - TGTTGCGGTATTTGTTGAGTT-3′ (antisense)), siME3-2 ((5′-CCAAGAUUACGACGACCUUTT-3′ (sense) and 5′ - AAGGTCGTCGTAATCTTGGTT-3′ (antisense)), and siME3-3((5′-GUGACCUGGACAAGUACAUTT-3′ (sense) and 5′ - ATGTACTTGTCCAGGTCACTT-3′ (antisense)). IPEC-J2 cells were transfected with either the NC siRNA or ME3 siRNA for 6 h. An aliquot of the transfected cells was collected to measure *ME3* gene expression, while another aliquot was treated with 0 mmol/L, 0.1 mmol/L, or 1 mmol/L MA for subsequent experiments.

### Real-time PCR

The absolute PCR experiment was conducted according to previously described methods [26]. DNA was extracted from the cecal contents using a commercial kit (D3141-03, Magen, Guangzhou, China). Briefly, 100 mg of contents was added to glass beads along with buffer ATL/PVP-10 and buffer PCI, then vortexed for 10 min to disrupt the samples. After heating and centrifugation, supernatant was incubated with RNase A, proteinase K, and AL buffer, then heated again. Anhydrous ethanol was added, and the mixture was passed through a HiPure DNA Mini Column II and centrifuged. The DNA was washed with Buffer GW1 and Buffer GW2, and finally the DNA was dissolved in nucleic acid-free water and stored at −80 °C.

To establish a standard curve, DNA was amplified via polymerase chain reaction (PCR, Biosystems QuantStudio 3, Takara, Japan). The reaction protocol consisted of an initial denaturation at 95 °C for 60 s, followed by 40 cycles of denaturation at 95 °C for 15 s, annealing at 60 °C for 60 s, and extension at 72 °C for 30 s. A final cycle of 95 °C for 15 s, 60 °C for 60 s, and 95 °C for 30 s was performed to obtain a melting curve. The PCR products were then recovered using a HiPure Gel Pure DNA Mini Kit (D2111-02, Magen). After separating the products in 1% agarose gel, the gel was excised and placed in a centrifuge tube. GDP buffer was added, and the mixture was heated at 55 °C for 15 min. The resulting mixture was transferred to a HiPure DNA Mini Column, centrifuged at 12,000 × *g* for 60 s, and cleaned with DW2 buffer. The purified DNA was dissolved in nuclease-free water and stored at −80 °C. To generate the standard curve, serial dilutions of DNA (10-, 100-, 1,000-, 10,000-, 100,000-, 1,000,000-, 10,000,000-fold dilutions) were prepared. The PCR reaction mixture (20 μL) contained 0.5 μL each of forward and reverse primers (10 μmol/L), 10 μL TB Green® Premix Ex Taq™ II (Tli RNaseH Plus) (Takara, Japan), 2 μL template DNA, and 7 μL ddH_2_O. Bacterial copy numbers were calculated using the corresponding standard curve, as described previously [27]. Briefly, the gene copy numbers were determined using the following equation: (DNA concentration (μg/μL) × 6.0233 × 10^23^ copies/mol)/(DNA size (bp) × 660 × 10^6^).

A real-time PCR assay was conducted as described previously [28]. Total RNA from cecal mucosa was extracted using the HiPure RNA Kit (R4130, Magen). Samples were treated with MagZol reagent and chloroform to obtain a nucleic acid supernatant. Ethanol was added to create suitable binding conditions, after which the mixture was transferred to the purification column and centrifuged. The RNA, bound to the membrane, was washed and then eluted with RNase-free water. One μg total RNA was reverse-transcribed into complementary DNA (cDNA, A0010CGQ, EZB). Briefly, the reaction system was: 1 μg total RNA, 5 μL 4 × RT Master mix, and nuclease-free ddH_2_O_2_ to a final volume of 20 μL. The reaction protocol was 42 °C for 15 min, followed by 95 °C for 30 s. SYBR Green relative quantitative real-time PCR was performed according to published protocols [29]. The reaction mixture (20 μL) contained 0.5 μL each of forward and reverse primers (10 μmol/L), 10 μL 2 × SYBR Green Master Mix (ROX2 Plus) (A0001-R2, EZB), 2 μL template DNA, and 7 μL ddH_2_O. The reaction protocol consisted of initial denaturation at 95 °C for 60 s, followed by 40 cycles of denaturation at 95°C for 15 s and annealing/extension at 60 °C for 60 s. A final cycle of 95 °C for 15 s, 60 °C for 60 s, and 95 °C for 30 s was performed to generate a melting curve. Results were calculated using the 2^−ΔΔCT^ method [26] and normalized to the expression of the housekeeping gene: *β-actin* for* Gallus* using, and *GAPDH* for *Sus*. The primer sequences are shown in Table 1.

**Table 1:** Primers used for measurement of gene expression level by quantitative PCR

Species	Gene	Primer sequences (5′→3′)	Amplicon size, bp	Accession number
*Gallus*	*SLC25A6*	F: TGCCAGATCCCAGAAACACT R: AGACCATGCACCCTTGAAGA	224	NM_204231
	*ATP6V0A1*	F: CCGTAGCTGACCTCGATTCT R: CACAGCAAACAGAAACGGGA	239	XM_046933240
	*UCP3*	F: GAGAAACAGAGCGGGATTTGAT R: GCTCCTGGCTCACGGATAGA	90	NM_204107
	*DAO*	F: TAAACTGCACTGGGATCCGT R: TAGATGCCTCCCAAAACCGT	190	XM_046928381
	*H6PD*	F: CCGCACCAGTTTCTATGAGC R: TACGCCTGATACTGACCCAC	201	XM_015297146
	*GLUD2*	F: GGTGCCAATGGACCTACAAC R: GTTCCACCATGCTTCCCAAA	242	XM_046920002
	*GPx1*	F: CGGCTTCAAACCCAACTTCA R: AAGTTCCAGGAGACGTCGTT	183	NM_001277853
	*HIF-1α*	F: TGAACTGCGCACACAATTCA R: CCAGGGAGTTGAGCGTATGA	185	NM_001396327
	*IDH1*	F: AAATGGCGCTGTCTAAAGGC R: TCATAGTTCTTGCAGGCCCA	226	XM_004942682
	*IDH2*	F: GACGGAGTTCGACAAGCTGA R: GTTCTTACACGCCCAGACGA	106	NM_001031599
	*ME1*	F: CCTACGTGTTCCCTGGAGTT R: GGTGGTGGCAGTGTTATTCC	224	NM_204303
	*ME3*	F: CTGAGGGCCGGGGTATATTT R: ATCAGAGATGTGCCGGACTC	155	XM_046902271
	*MTHFD2*	F: CAACGTGGATGGCCTGTTAG R: AGCATTGCAAATCTTCCGCT	70	NM_001031360
	*NNT*	F: TGAAGCCGAAGACTGTAGCA R: TGAGTGGAGAGTGAAGAGCG	234	XM_015277552
	*NOX4*	F: GCAGGGACGTCGAATCTTTC R: GTCGTCCGATCAAAAGCCTC	183	NM_001101829
	*LOC107050147*	F: CACCCCAAACACATCGGGA R: AAAGCCGGCCTTGAAGAG	222	XM_040653270
	*PPARγ*	F: CCAGCGACATCGACCAGTTA R: CTTGCCTTGGCTTTGGTCAG	109	XM_046925952
	*PGD*	F: TAGTGCCGTTGTTGGAGACT R: TGGCATGAGTGAAGGACCAT	167	NM_001006303
	*β-Actin*	F: CTGTGCCCATCTATGAAGGCTA R: ATTTCTCTCTCGGCTGTGGTG	139	NM_205518
*Sus*	*DUOX2*	F: CTCTGCTGACTGTACCCCTG R: GCTCGTGTTGTCTCAGGTTG	108	NM_213999
	*GAPDH*	F: GTCGGAGTGAACGGATTTGGC R: CACCCCATTTGATGTTGGCG	250	NM_001206359
	*GPx4*	F: TGGCCTCTCAATGAGGCAAG R: CCCTTGGGCTGGACTTTCAT	253	NM_214407
	*ME3*	F: GAACAAGCTCTCCAACCACG R: TTCTCATGGTTCAGGTGGCT	186	NM_001244258
	*NOX4*	F: ACAACTGTTCCTGGCCTGAC R: CATCCTGGTAGTGCGTTCCA	241	XM_013979249
All bacteria (target V3–V4 region)		F: ACTCCTACGGGAGGCAGCAG R: ATTACCGCGGCTGCTGG	200	

### Biochemical analysis

Serum, cecal contents, and mucosa were collected. Serum was used for testing without special handling. The following enzyme activities and contents were detected using commercial kits: superoxide dismutase (SOD) (BC0170, Solarbio), oxidized thioredoxin reductase (TRX) (BC1155, Solarbio), glutamate dehydrogenase (GDH) (BC1460, Solarbio), glutathione peroxidase (GPx) (S0057S, Beyotime), ROS (BB470515, Bestbio), total antioxidant capacity (T-AOC) (A015-3-1, Nanjing jiancheng), malonaldehyde (MDA) (S0131S, Beyotime), H_2_O_2_ (S0038, Beyotime), nicotinamide adenine dinucleotide phosphate (NADPH) (S0038, Beyotime), reduced glutathione (GSH) (BC1175, Solarbio), reduced glutathione (GSSG) (BC1185, Solarbio), and Chromogenic LAL Endotoxin Assay Kit (Lipopolysaccharide, LPS) (C0276S, Beyotime).

Nine volumes of methanol were added to the contents based on the weight. The mixture was then homogenized and centrifuged according to specific instructions for each assay. Cells were lysed using a radioimmunoprecipitation assay (RIPA) buffer containing phenylmethylsulfonyl fluoride (PMSF) (BB3201, Bestbio). Unless otherwise indicated, all assays were corrected using either Bicinchoninic Acid Assay (BCA) (23227, Thermo) or sample weight. All procedures were performed according to the manufacturer’s instructions.

### 16S rRNA

Cecal contents were used for 16S rRNA. Library construction was carried out following the protocol of the ALFA-SEQ DNA Library Prep Kit (NDI001E-01, Findrop), and the size of the library fragments was evaluated on the Qsep400 High-Throughput Nucleic Acid & Protein Analysis System (Hangzhou Houze Biotechnology Co., Ltd., China). The concentration of the library was measured using a Qubit 4.0 (Thermo Fisher Scientific, Waltham, MA, USA). The primer sequences were 338F: 5′-ACTCCTACGGGAGGCAGCA-3′ and 806R: 5′-GGACTACHVGGGTWTCTAAT-3′, targeting V3–V4 region. The constructed amplicon libraries were subjected to PE250 sequencing on either the Illumina or MGI platform (Guangdong Magigene Biotechnology Co., Ltd., Guangzhou, China).

A similarity of 97% was clustered into operational taxonomic units (OTUs). In addition, we excluded the influence of low-abundance species and retained OTUs with relative abundance > 0.01%. For bioinformatics analysis, the bacterial diversity index (Simpson and Shannon), richness (ACE and Chao1), bacterial community structure of principal coordinates (PCoAs), and linear discriminant analysis effect size (LEfSe) analysis were performed. LEfSe analysis was used to calculate significance based on linear discriminant analysis (LDA) effect size ≥ 4.5 and *P* < 0.05. In addition, we also analyzed the proportions of Firmicutes and Bacteroidetes of each group at the phylum level, and Firmicutes/Bacteroidetes at the phylum level was made.

### Statistics and data analysis

Statistical analyses were performed using GraphPad Prism 8.0 software (Chicago, IL, USA). Differences between the two groups were assessed using the *t*-test. One-way analysis of variance (ANOVA) was used for comparisons between multiple groups. Duncan’s multiple range test was used to compare the differences among groups. *T*-test and Wilcox Rank-Sum test were used for α diversity analysis. R (version 3.2.0) software was used for β diversity analysis. All fluorescent images were calculated using the Image J. The data were presented as the mean ± SEM. *P* ≤ 0.05 was regarded as statistically significant.

## Results

### Cecal redox and energy metabolites in the growth performance of broilers

**Fig. 1:**
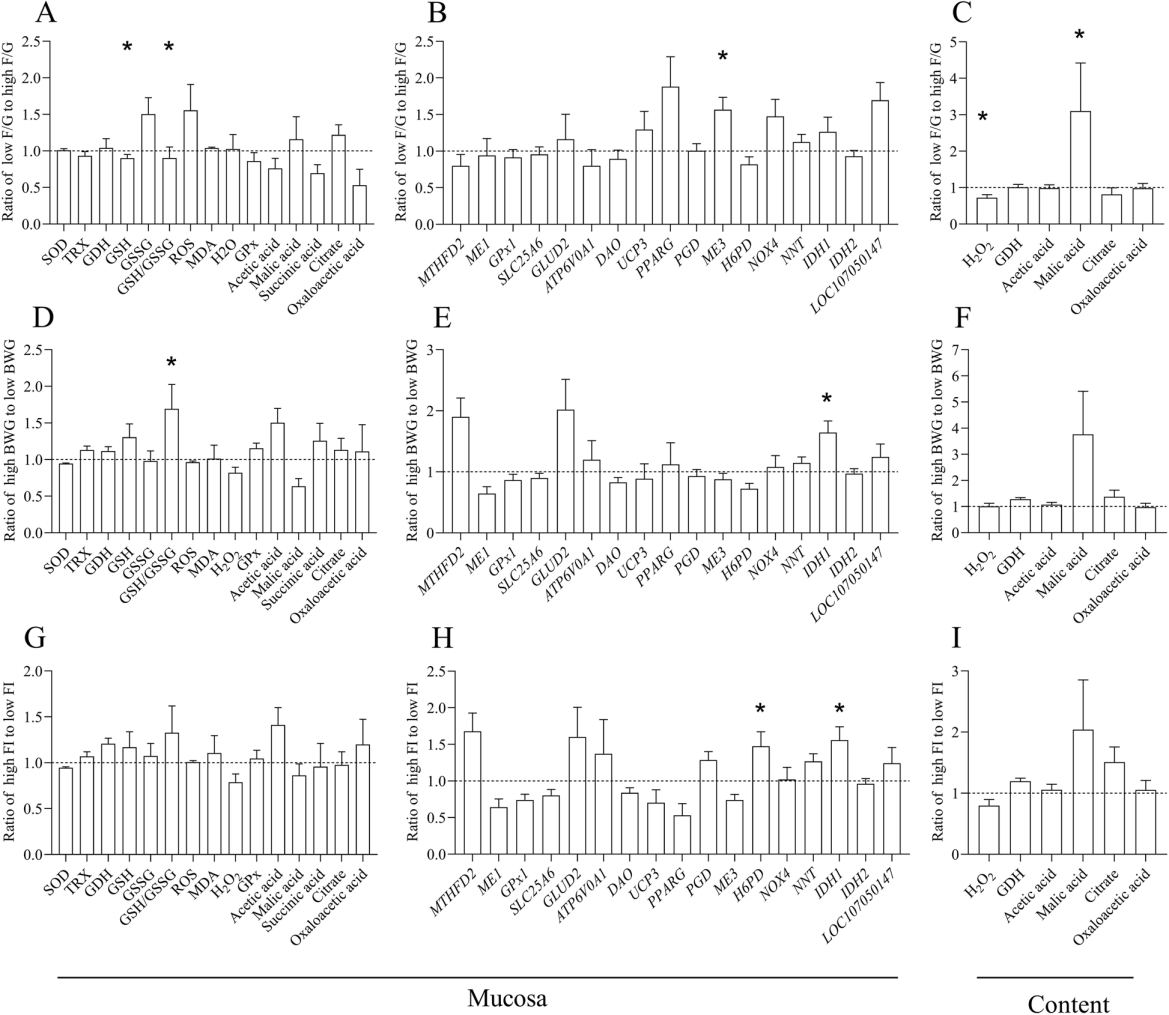
Cecal redox and energy metabolites involved in the growth performance of broilers. **A–C** Differences metabolites between high and low F/G in cecum. **D**–**F** Differences metabolites between high and low BWG in cecum. **G**–**I** Different metabolites between high and low FI in cecum. *T*-test was used, and error bars represented the SEM (*n* = 15), * indicates a difference of *P* ≤ 0.05

To explore the differences in redox and energy metabolites in the cecum tract, in trial 1, we analyzed mRNA expression in the cecal mucosa and metabolites in both cecal mucosa and contents. We compared values between two groups with different feed efficiency. The results demonstrated that the cecal mucosal GSH, *ME3* mRNA (approximately 1.5-fold) expression, and MA in content (approximately 3-fold) with a low F/G ratio were higher than those with a high F/G ratio. (Fig. 1A–C). GSH/GSSG (approximately 1.7-fold) and *IDH1* mRNA (approximately 1.5-fold) were increased in high BWG (Fig. 1D–F). Moreover, *H6PD* and *IDH1* mRNA (approximately 1.5-fold) were higher in high FI (Fig. 1G–I). Finally, our analysis focused on indicators of F/G. The redox capacity with low F/G was enhanced in broilers.

### Colonic perfusion of MA alleviates colitis in mice

**Fig. 2:**
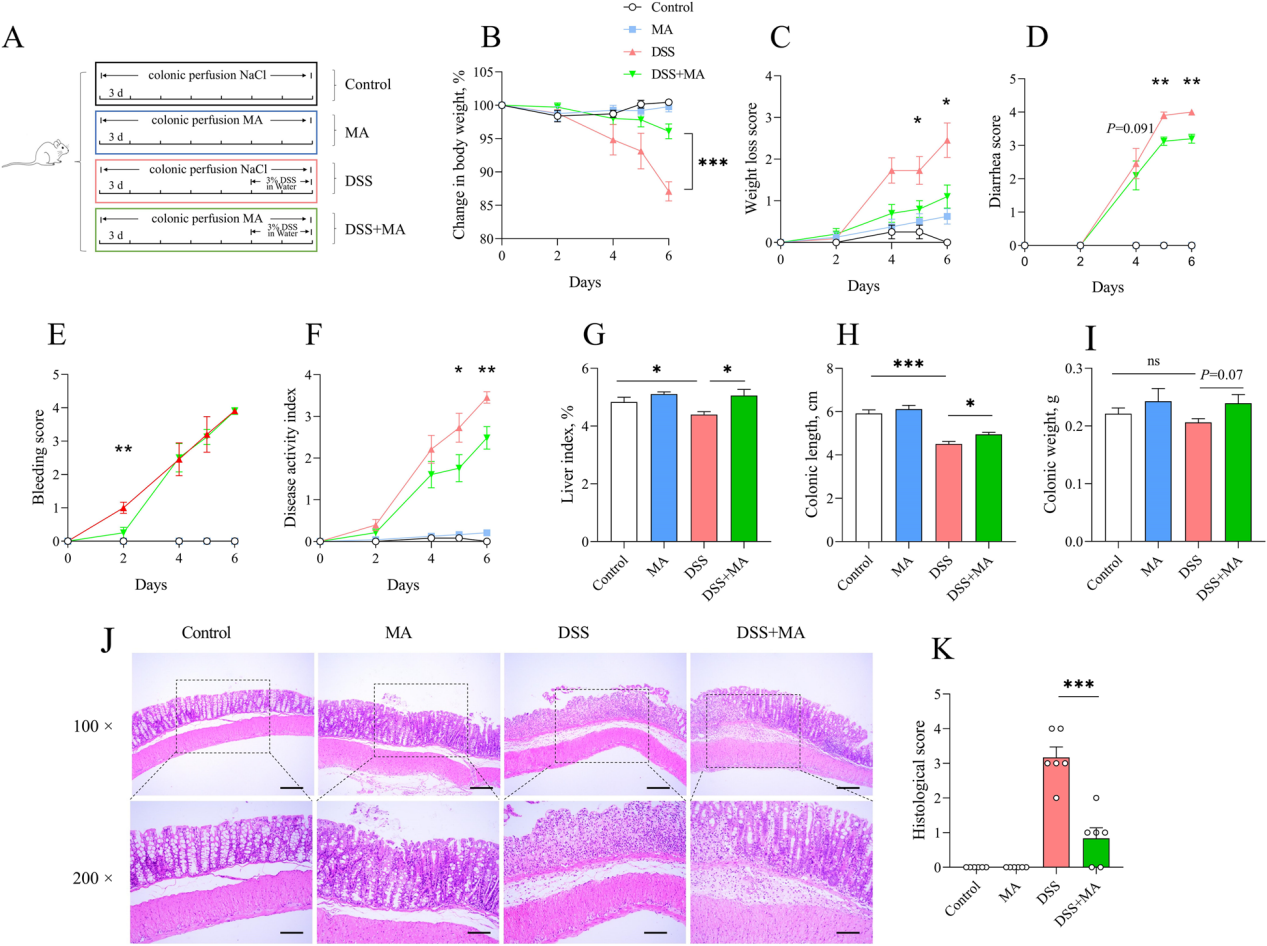
Colonic perfusion of MA alleviates colitis in mice. **A** Schematic diagram of DSS-treated mice. **B** and **C** Percentage weight loss and weight loss score. **D–F** Diarrhea, fecal occult blood score and DAI. **G**–**I** Liver index, colon length and weight. **J** and **K** H&E staining (100 × with a scale of 100 μm and 200 × with a scale of 50 μm) and histopathological scores. The values are presented as the mean ± SEM (*n* = 8). *, ** and *** indicate differences of *P* ≤ 0.05, *P* ≤ 0.01, and *P* ≤ 0.001, respectively

To investigate the effects of MA on intestinal oxidative stress and ulcerative colitis, we employed a dextran sulfate sodium (DSS)-induced colitis mouse model with colon perfusion of MA. The results showed that MA administration mitigated several symptoms in DSS-induced colitis mice, including body weight loss, weight loss score, diarrhea score, and DAI in DSS-induced colitis mice, but had no significant impact on fecal occult blood (Fig. 2B–F). MA administration counteracted the detrimental effects of DSS on the liver index, colon length and weight (Fig. 2G–I), as well as the inflammatory cell infiltration in the colon (Fig. 2J and K).

### Colonic perfusion MA reduces oxidative stress and inflammation and increases the intestinal barrier in mice

**Fig. 3:**
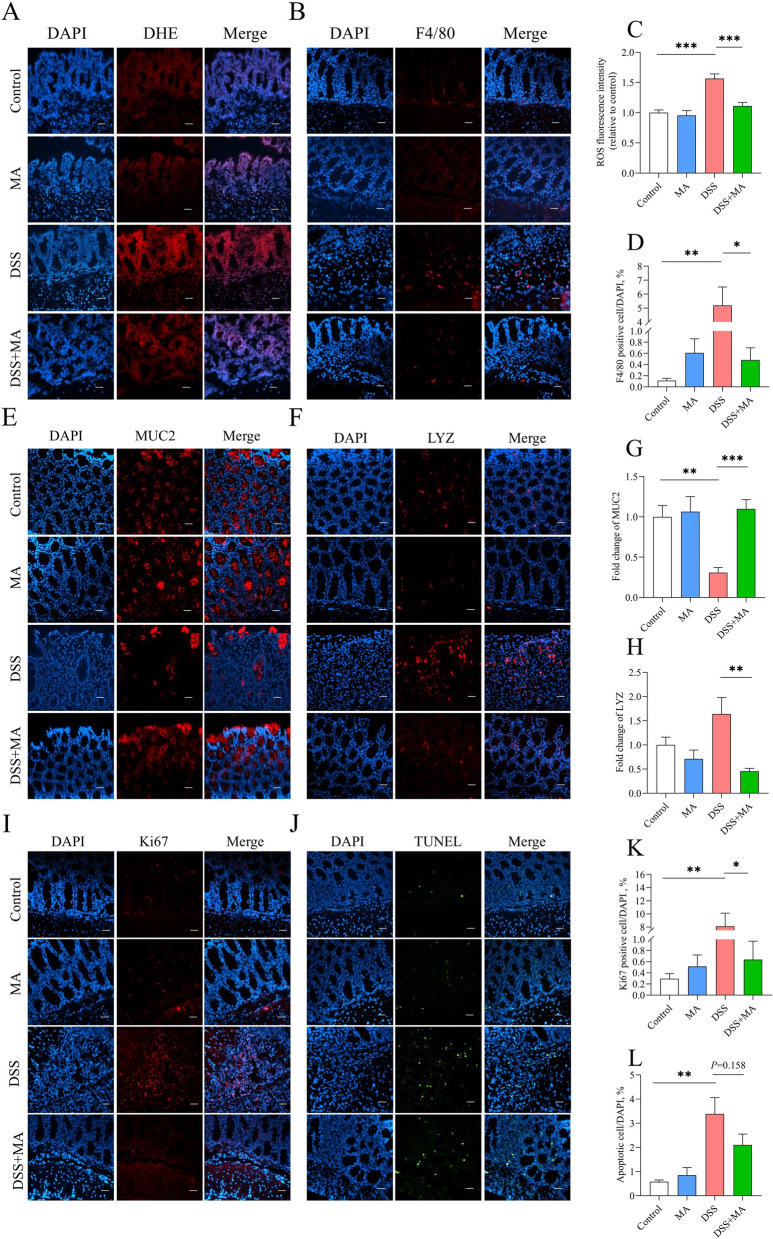
Colonic perfusion of MA alleviates colitis in mice. **A**–**D** Colonic ROS fluorescence intensity and statistical plots (relative to the control), colonic F4/80 counts and statistics. **E**–**H** MUC2 content and statistical plots (relative to the control), paneth cell counts and statistics (relative to the control). **I**–**L** Proliferating cell counts and statistics of colonocytes, apoptotic cell counts and statistics of colonocytes. All the immunofluorescence magnifications are 400 × , and the scale is 50 μm. The values are presented as the mean ± SEM (*n* = 8). *, ** and ***indicate differences of *P* ≤ 0.05, *P* ≤ 0.01, and *P* ≤ 0.001, respectively

To assess oxidative stress and the intestinal barrier function, we performed ROS, F4/80, MUC2, and LYZ staining on the colon. MA administration significantly reduced DSS-induced oxidative stress and macrophages infiltration (Fig. 3A–D), while enhancing the intestinal barrier of the colon (Fig. 3E–H). In addition, colitis typically leads to abnormal proliferation and apoptosis of intestinal cells. Here, we found that colonic infusion of MA reduced cell proliferation, but did not affect on apoptosis caused by colitis (Fig. 3I and L).

### Effects of dietary supplementation with MA and MAP on the growth performance of broilers

**Fig. 4:**
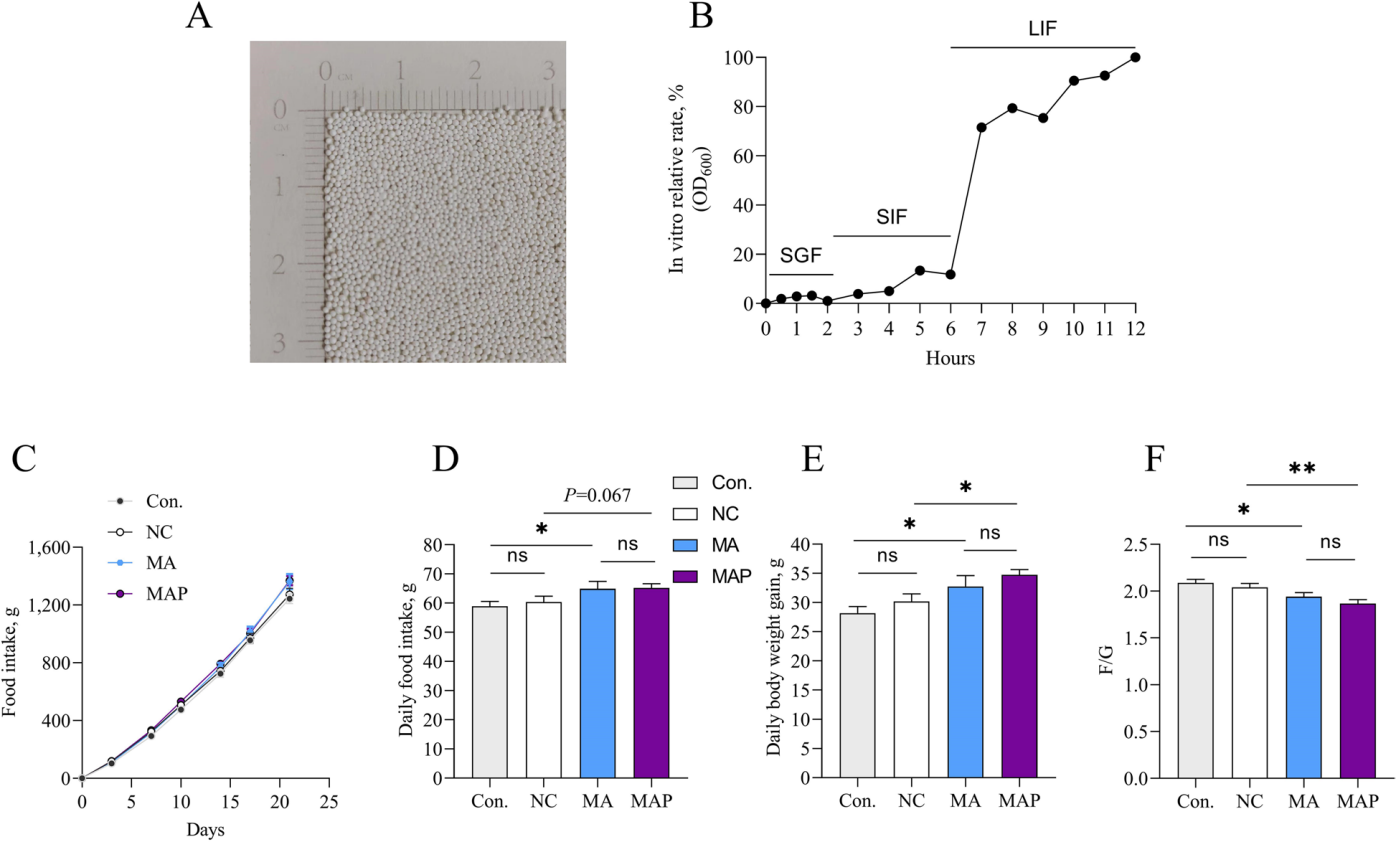
Effects of dietary supplementation with MA and MAP on the growth performance of broilers. **A** Illustration of pellet size. **B** Release test of blank pellets in vitro. **C**–**F** The 21–45 d cumulative feed intake (**C**), average daily feed intake (**D**), average daily gain (**E**) and F/G (**F**) of broilers. The values are presented as the mean ± SEM (*n* = 30). * and ** indicate differences of *P* ≤ 0.05 and *P* ≤ 0.01, respectively

To achieve spatial-specific release in the hindgut of broilers, we developed a coated pellet for controlled release. The release curve showed that the pellet release rate was less than 10% in both simulated gastric fluid (SGF) and artificial small intestine fluid (SIF), but achieved complete release in large intestine fluid (LIF) (Fig. 4A and B). We then supplemented diets with MA and MAP to investigate broiler growth performance. We observed that the MA and MAP increased average daily feed intake by approximately 5 g/d compared to the Con. Moreover, MA and MAP increased average daily body gain more than Con, with MAP outperforming MA by 2 g/d. In addition, MA and MAP reduced F/G compared to the Con, with MA showing a lower F/G than Con, and MAP demonstrating an even lower F/G than MA by 0.1. Thus, MAP exhibited a better effect than MA on broiler growth performance (Fig. 4C–F).

### Effects of dietary MAP supplementation on the slaughter performance and colon length in broilers

We further investigated the effects of MAP on broiler slaughter performance and organ indices. While no differences were observed in liver weight, MAP significantly increased the slaughter weight, eviscerated carcass weight, abdominal fat weight, breast and thigh muscle weight, and colon length (Table 2). MAP and MA groups had slaughter weight over 100 g heavier than Con, with MAP outweighing MA by more than 40 g. For eviscerated carcass weight, both MAP and MA groups were over 100 g heavier than Con, with MAP exceeding MA by more than 20 g. Abdominal fat weight in MAP and MA groups was more than 2 g heavier than the Con, with MAP surpassing MA by an additional 2 g. Breast muscle weight in MAP and MA groups was more than 14 g heavier than Con. The thigh muscle weight in the MAP group was 18 g higher than Con and 5 g higher than the MA group. Additionally, the colon length in the MAP group was 0.9 cm longer than Con.

**Table 2:** Effects of dietary MAP supplementation on the slaughter performance and organ indices of broilers

Items	Con	NC	MA	MAP	*P*-value
Slaughter weight, g	894.23 ± 16.32^b^	909.92 ± 22.51^b^	1,026.40 ± 40.77^a^	1,066.30 ± 33.959^a^	< 0.001
Eviscerated carcass weight, g	640.70 ± 13.03^b^	654.00 ± 18.62^b^	750.68 ± 33.32^a^	772.19 ± 24.79^a^	< 0.001
Abdominal fat weight, g	7.85 ± 0.72^b^	7.36 ± 1.04^b^	9.71 ± 1.06^ab^	11.77 ± 1.09^a^	0.011
Breast muscle weight, g	63.74 ± 2.29^b^	68.58 ± 3.00^b^	77.77 ± 3.85^a^	77.48 ± 2.89^a^	0.002
Thigh muscle weight, g	58.48 ± 1.70^b^	59.90 ± 2.02^b^	70.97 ± 3.85^a^	76.39 ± 2.79^a^	< 0.001
liver weight, g	26.08 ± 1.04	26.35 ± 1.53	26.17 ± 0.97	27.64 ± 1.17	0.820
Colon length, cm	5.61 ± 0.22^b^	5.73 ± 0.21^b^	6.32 ± 0.23^a^	6.48 ± 0.2^a^	0.002

^a,b^Means with different lowercase letters in each row are statistically significantly different (*P* ≤ 0.05). The values are presented as the mean ± SEM (*n* = 15)

### Dietary supplementation of MAP improved the cecal redox capacity and the TCA cycle of broilers

**Fig. 5:**
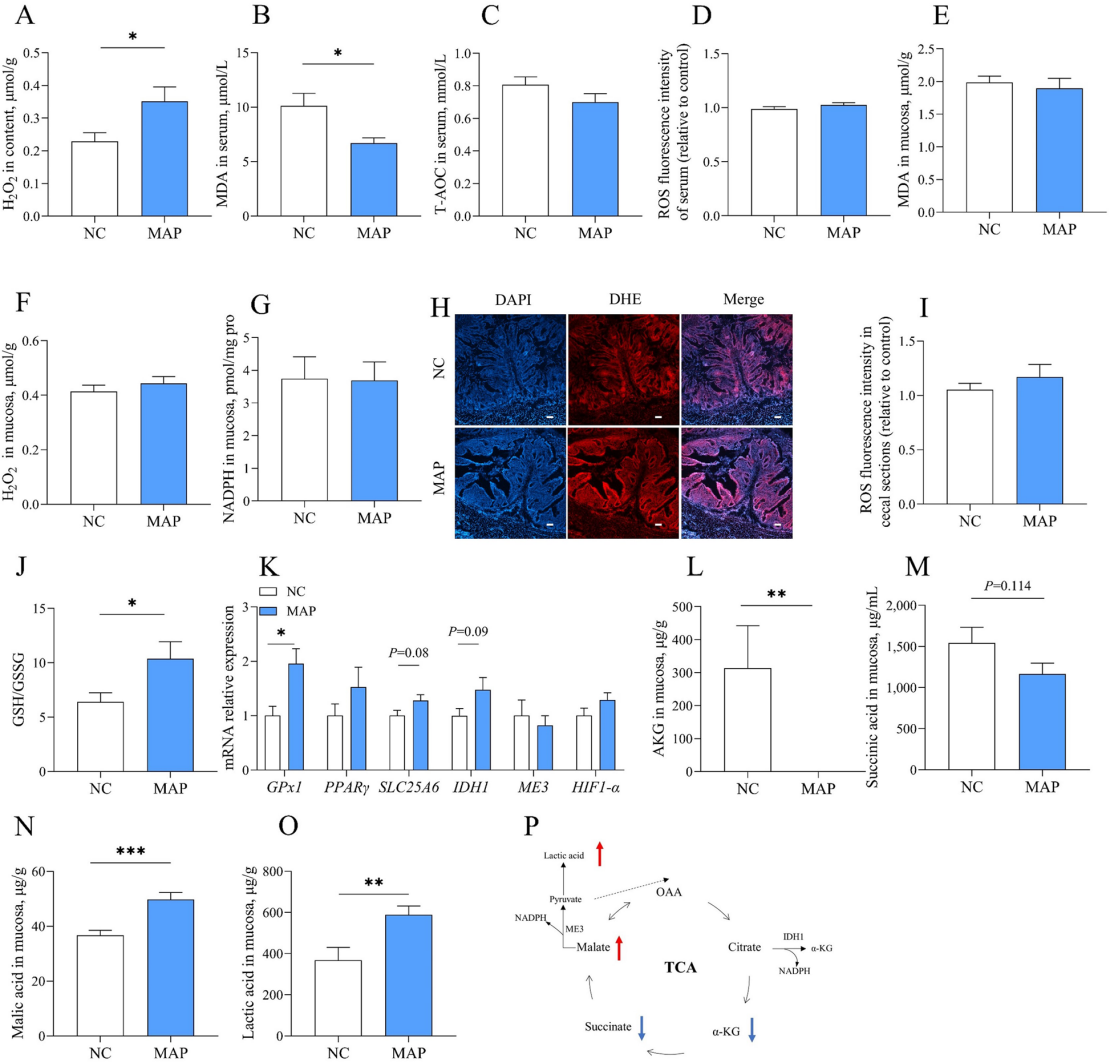
Dietary supplementation of MAP improved the cecal redox capacity and the TCA cycle of broilers. **A** H_2_O_2_ in cecal contents. **B**–**D** Serum MDA (**B**), ROS (**C**) and T-AOC levels (**D**). **E**–**I** Mucosal MDA (**E**), H_2_O_2_ (**F**), NADPH (**G**), cecal DHE staining (**H**) and ROS fluorescence intensity (**I**). **J** Mucosal GSH/GSSG ratio. **K** mRNA expression in redox. **L**–**O** Mucosal AKG (**L**), succinic acid (**M**), MA (**N**) and lactic acid (**O**). **P** Diagram of the mechanism of MA in the TCA cycle. The values are presented as the mean ± SEM (*n* = 10). * and ** indicate differences of *P* ≤ 0.05, and *P* ≤ 0.01, respectively

We further investigated the effect of MAP on the redox status of the cecum in broilers. The results showed that MAP significantly increased the H_2_O_2_ in cecal content (Fig. 5A). Moreover, MAP lowered serum MDA level but had no effect on the serum ROS level or total antioxidant capacity (T-AOC) (Fig. 5B–D). While MAP had no impact on mucosal MDA, H_2_O_2_, NADPH, or intestinal ROS contents (Fig. 5E–I), it markedly elevated the GSH/GSSG ratio and *GPx1* mRNA expression were markedly elevated (Fig. 5J and K). In the cecal mucosa, MAP decreased AKG and succinic acid level while raising MA and lactate level (Fig. 5L–O).

### Dietary MAP supplementation changes the intestinal microbiota of broilers

**Fig. 6:**
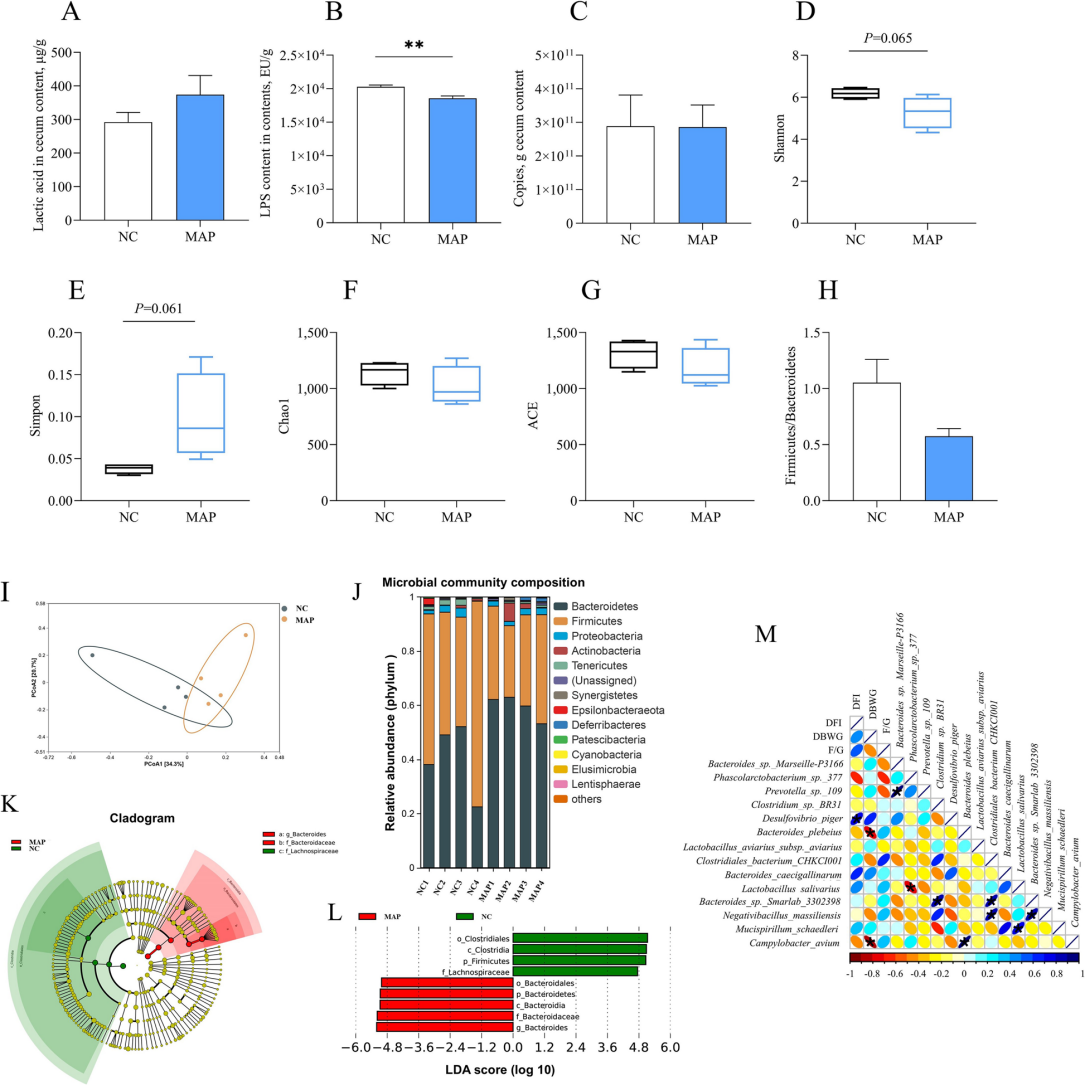
Dietary MAP supplementation changed the intestinal microbiota of broilers. **A** Lactic acid contents in content. **B** LPS contents in content. **C** Bacterial numbers of contents. **D**–**H** Shannon, Simpson, Chao1, ACE, Firmicutes/Bacteroidetes. **I–L** PCoA, microbial community composition, cladogram and LDA score. **M** Correlation analysis of growth performance and intestinal microbiota (species level). The values are presented as the mean ± SEM (*n* = 15). ** indicates difference of *P* ≤ 0.01

To determine the effects of MAP on intestinal microbiota, the metabolites in cecum contents was measured. We found that MAP had no significance on lactic acid level, but reduced total LPS in the contents (Fig. 6A and B). Moreover, the total number of bacteria was not different between the groups (Fig. 6C). 16S rRNA sequencing analysis revealed that in MAP group Shannon index decreased and Simpson index increased, while Chao1 index and ACE index had no significance (Fig. 6D–G). Thus, the α diversity of the content microbiota showed a slightly significant difference between the two groups. Moreover, β diversity of PCoA revealed that the bacterial composition of MAP was distinct from NC (Fig. 6I). At the phylum level, NC had a higher relative abundance of Firmicutes and a lower relative abundance of Bacteroidetes compared to MAP. Consequently, the Firmicutes/Bacteroidetes ratio was decreased in MAP (Fig. 6H and J). Meanwhile, we noticed that Bacteroides and Bacteroidaceae had an important role in MPA, while Lachnospiraceae had a pivotal role in NC (Fig. 6K). LDA score also confirmed this point. Moreover, Clostridiales, Clostridia, Firmicutes and Lachnospiraceae in NC significantly different compared with MAP (Fig. 6L). Correlation analysis showed that Bacteroides in MAP group had positive correlation with DFI (Fig. 6M).

### ME3 is essential for MA to enhance the redox capacity of intestinal epithelial cells

**Fig. 7:**
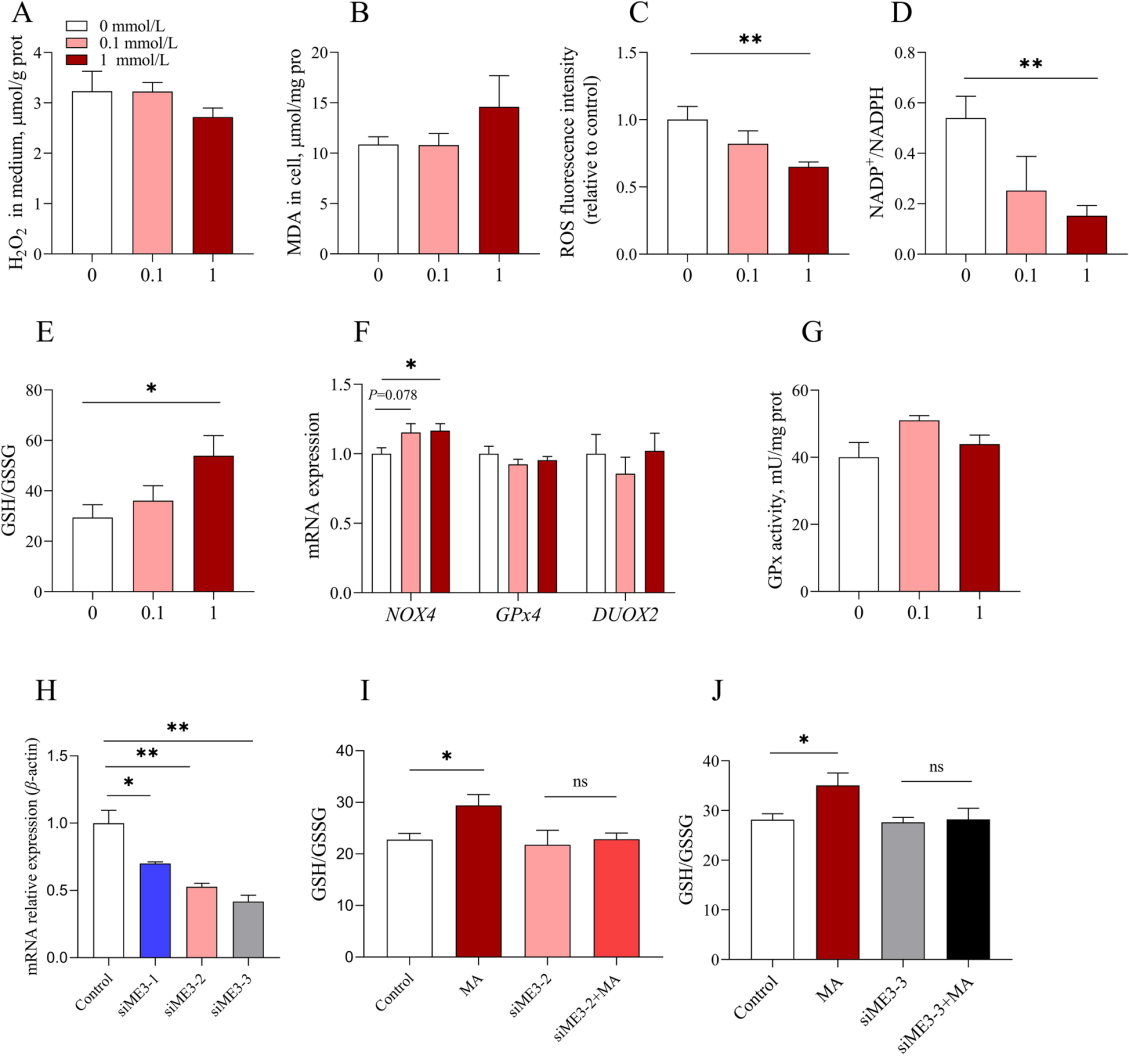
ME3 is essential for MA to enhance the redox capacity of intestinal epithelial cells. **A** H_2_O_2_ in medium. **B** Intracellular MDA content. **C–E** Intracellular ROS fluorescence intensity (relative to the control), NADP^+^/NADPH ratio and GSH/GSSG ratio. **F**–**G** GPx enzyme activity and mRNA expression in related-genes of redox. **H**–**J** Interference efficiency and GSH/GSSG ratio. The values are presented as the mean ± SEM (*n* = 6). * and ** indicate differences of *P* ≤ 0.05, and *P* ≤ 0.01, respectively

To explore the role of MA in redox capacity, we treated IPEC-J2 cells with various concentrations of MA. Initially, we first found that MA had no effect on intracellular H_2_O_2_ or MDA levels. (Fig. 7A and B). However, MA significantly reduced intracellular ROS level and NADP^+^/NADPH ratio, while increasing the GSH/GSSG ratio (Fig. 7C–E). Similarly, MA had no effect on GPx enzyme activity, *GPx4* or *DUOX2* mRNA expression, but significantly increased *NOX4* mRNA expression (Fig. 7F and G). Based on our observation of higher *ME3* mRNA levels in low F/G groups in trial 1, we investigated the function of *ME3* in redox capacity. We interfered with *ME3* and co-treated cells with MA. The result demonstrated that co-treatment decreased the intracellular GSH/GSSG ratio after two different siME3 knockout (Fig. 7H–J).

## Discussion

Numerous studies have shown that oxidative stress negatively affects feed efficiency [30–32]. It is the main cause of reduced FE in broilers, disrupting the redox system and energy metabolism. Birds adapt to oxidative stress by increasing the levels of endogenous antioxidants [33]. In trial 1, we screened the main metabolite indicators, including energy metabolism and redox, in the cecum of broilers. We found that broilers with lower F/G had higher GSH, GSH/GSSG, and MA contents in the cecal chyme. *ME3* and *H6PD* mRNA expression were higher in the low F/G and high FI groups. *IDH1* mRNA expression was higher in the high BWG and high FI groups. These findings indicated that enhanced redox capacity in low F/G broilers.

In the redox balance system, both *ME3*, *H6PD*, and *IDH1* are required for NADPH production [34, 35]. NADPH serves two major functions: generating superoxide (O^2−^) by NADPH oxidases (NOXs) and the scavenging of H_2_O_2_ by regenerating GSH and the TRX [36]. MA, a C4 dicarboxylic acid, is primarily produced through microbial fermentation and enzymatic synthesis [37]. It has been reported that MA has pivotal role of maintaining high level of GSH, thereby reducing oxidative stress and inflammation [38, 39]. Interestingly, we also noticed that SOD and GPx had no significance between high and low growth performance. The redox system exhibits tissue specificity and age-related differences, responding variably to diverse stress challenges. For example, SOD and GPx are crucial in the livers of aged rats and the hypothalamus of stressed rats, but not in the heart [40, 41]. Under heat stress, 21 d broilers demonstrated greater antioxidant capacity than 45 d broilers [42]. These evidences suggest that the MA/*ME3* pathway, but not SOD or GPx, might be specifically important for hindgut redox homeostasis. However, MA originates from the citrate pyruvate cycle and oxaloacetate, and is influenced by various enzymes. An increase in MA elevates *ME3* expression, while elevated *ME3* expression leads to a decrease in MA. Consequently, changes in MA and *ME3* levels may not occur synchronously.

Oxidative stress is considered a major cause of intestinal inflammation. Its destructive effects may contribute to the initiation and/or propagation of the inflammatory bowel diseases [43]. Interestingly, several studies have reported increased levels of MA during colitis treatment [44, 45]. To reveal the effect of MA on intestinal inflammation, a DSS-induced colitis mouse model was adopted with colon-perfused of MA. We found that MA significantly reduced weight loss, diarrhea score, and DAI. However, after DSS induction, the liver index, colon length, and weight were significantly increased. We also noticed that the colon length was longer in the MAP group. Additionally, MA significantly reduced the colonic histopathological score. Colitis typically presents with goblet cell reduction, a thinner mucus layer, immune cell infiltration, abnormal and persistent crypt cell hyperproliferation, and increased apoptosis [46–48]. We found that MA significantly decreased the colonic ROS, macrophage infiltration, Panzer cell number, and abnormal colonic proliferation, while increasing goblet cell number. Recent research has showed that organoids produce MA during transplantation, which becomes enriched in cecal contents. MA regulates macrophage polarization and promotes intestinal recovery from ischemia–reperfusion (I/R) injury through a suppressor of cytokine signaling 2-dependent mechanism, positively impacting intestinal injury repair [38]. In addition, we observed that blood in the stool did not improve at the later stages. We hypothesized that as the duration of DSS induction increased, bleeding also occurred in the foregut, an area that MA did not reach. Therefore, these results indicate that MA could reduce colonic oxidative stress and inflammation, increase the intestinal barrier in mice, and consequently relieve colitis.

Intestinal inflammation leads to decreased feed intake, compromised intestinal health, and ultimately diminished growth performance in livestock [49]. Our previous research indicated that oxidative stress is more pronounced in the hindgut compared to the foregut [13]. Therefore, we used a controlled-release technique to deliver MA, which relieves intestinal inflammation in the hindgut. Here, we found that the MAP group exhibited a better daily feed intake, daily body weight gain and a lower F/G ratio than the MA group. It has been reported that the supplementation of 0.4% or 0.8% of MA to the diets of AA broilers at 2,242 d increased the BWG and decreased the F/G ratio [50]. However, supplementation of 0, 0.5%, 1% and 2% MA to the basal diet were found to had no effect on growth performance of fattening pig or Landrace × Yorkshire sows [51, 52]. By using an encapsulation technique, our lower dose of MA (1‰) achieved effects comparable to 0.4% MA supplementation. In addition, the MAP group demonstrated smaller within-group error and coefficient of variation compared to the other three groups. These findings suggest that individual differences in chicken farming may significantly contribute to hindgut oxidative stress. The encapsulation technique has gained widespread use in recent years [53], indicating its great potential in livestock and poultry breeding.

MA plays an important role in energy metabolism, redox balance and lipid metabolism. It serves as both an intermediate in the TCA cycle, catalyzed by malate dehydrogenase to produce oxaloacetate, and a substrate for MEs. MEs generate NADPH, which is vital for the scavenging free radicals in the redox system or producing ROS via NADPH oxidases [54, 55]. Here, MAP reduced serum MDA concentration and increased the mucosal GSH/GSSG ratio. Similarly, MA increased the GSH/GSSG ratio and decreased the NADP^+^/NADPH ratio in vitro. GSH/GSSG is the major redox pair in cells, with glutathione reductase catalyzing GSSG reduction to GSH using NADPH as an electron donor to regenerate GSH [56, 57]. These findings are consistent with previous reports that MA reduces the MDA level in rat liver and heart, increases the GSH content, and accelerates the elimination of the damage caused by ROS [40]. MA has multiple metabolic pathways in the cell. For example, in the mitochondrial matrix, malic acid can be catalyzed by malate dehydrogenase to produce oxaloacetate and NADH. It can also be metabolized by ME1 or ME3 to generate pyruvate and NADPH. Pyruvate can then be further converted to lactate or enter the TCA cycle [36]. Here, we speculate that the oxygen content in the hindgut is too low for MA to enter the TCA cycle and undergo oxidative phosphorylation. Thus, MA likely generates pyruvate, which is eventually converted to lactate. Notably, MAP increased H_2_O_2_ in the cecum content, whereas MA treatment failed to alter H_2_O_2_ levels in the cell medium. We speculate the divergence might be due to the intestinal microbiota. Moreover, NADPH oxidases (NOXs) are also a pathway for ROS production [8]. We also found that in IPEC-J2 cells, MA treatment of cells resulted in elevated mRNA expression of *NOX4*.

In trial 3, we discovered that MAP decreased the amount of LPS, so we made 16S rRNA. We found that MAP reduced bacterial α diversity, and the contribution of gut microbiota to the beneficial effects of MA was *Bacteroides*. *Bacteroides* are anaerobic and Gram-negative rods, which consume polysaccharides in the hindgut and produce short-chain fatty acids (SCFA) [58]. Previous research has revealed that fecal bacterial genera, such as *Bacteroides,* were more abundant in high feed conversion ratio chickens [59]. This is consistent with our findings. Therefore, we hypothesized that differences in the amount of SCFA in the cecum may contribute to FE variability. Moreover, previous studies in germ-free mice have shown that during hindgut development, *Bacteroides thetaiotaomicron* stimulates angiogenesis, which is associated with the formation of a capillary network that efficiently distributes absorbed nutrients [60]. The normal gut microbiota has also been shown to impart a healthy metabolome in the serum by increasing the concentrations of pyruvic acid, citric acid, fumaric acid and malic acid, all of which are indicators of higher energy metabolism [61]. However, the precise mechanisms by which MA regulates the interaction between bacteria and intestinal epithelial cells require further investigation.

Malic enzymes (MEs) play crucial roles in cellular energy production, redox homeostasis, and cancer development by converting MA to pyruvate and NADPH. Three ME isoforms have been identified: cytoplasmic NADP-dependent malic enzyme 1 (ME1), mitochondrial NAD(P)-dependent malic enzyme 2 (ME2) and mitochondrial NADP-dependent malic enzyme 3 (ME3) [35]. It has been well studied that knockdown of ME1 and ME2 can cause oxidative stress. Moreover, there is a compensatory relationship among MEs [62–64]. However, the role of ME3 had rarely been investigated. In this study, we found the positive relationship between *ME3* and a low F/G ratio, but not *ME1*. To reduce the off-target effect, we used two independent siRNA sequences for interference. It was found that both of them could effectively block the effect of MA on GSH/GSSG, suggesting that ME3 is essential for MA.

## Conclusion

The MA/*ME3* pathway-mediated redox capacity was closely related to the F/G ratio of broilers; targeted delivery of MA to the hindgut or colonic perfusion improved intestinal redox capacity, reduced inflammation, and enhanced feed efficiency.

## Supplementary Information

Additional file 1: Table S1. Composition of basal diet.

## Abbreviations

AA: Arbor acre
ACE: Abundance-based coverage estimator
ATP6V0A1: ATP synthase gamma
BWG: Body weight gain
DAI: Disease activity index
DAO: D-amino acid oxidase
DBWG: Daily body weight gain
DFI: Daily feed intake
DHE: Dihydroethidium
DSS: Dextran sulfate sodium salt
DUOX2: Dual oxidase 2
FE: Feed efficiency
F/G: Feed to gain
FI: Feed intake
GAPDH: Glyceraldehyde-3-phosphate
GLUD2: Glutamate dehydrogenase 2
GPx1: Glutathione peroxidase 1
GSH: Reduced glutathione
GSSG: Oxidized glutathione
H_2_O_2_: Hydrogen peroxide
H6PD: Glucose-6-phosphate
HIF-1α: Hypoxia-inducible factor α
IDH1: Isocitrate dehydrogenase 1
IDH2: Isocitrate dehydrogenase 2
IPEC-J2: Porcine intestinal epithelial cell
LDA: Linear Discriminant Analysis
LIF: Large intestine fluid
LPS: Lipopolysaccharide
LOC107050147: Pyruvate dehydrogenase kinase 2
MA: Malic acid
MAP: Malic acid pellet
MDA: Malonaldehyde
ME1: Malic enzyme 1
ME2: Malic enzyme 2
ME3: Malic enzyme 3
MTHFD2: Methylenetetrahydrofolate
NADPH: Nicotinamide adenine dinucleotide phosphate
NNT: Nicotinamide nucleotide
NOX4: Nicotinamide adenine
PCoA: Principal Coordinate Analysis
PPARγ: Peroxisome proliferator-activated receptor γ
ROS: Reactive oxygen species
SCFA: Short-chain fatty acids
SGF: Simulated gastric fluid
SIF: Artificial small intestine fluid
SLC25A6: Adenine nucleotide translocase
SOD: Superoxide dismutase
T-AOC: Total antioxidant capacity
TRX: Oxidized thioredoxin reductase
UCP3: Avian uncoupling protein 3
β-actin: Actin-beta
