# Supplementary material for: Malic enzyme 3 mediated the effects of malic acid on intestinal redox status and feed efficiency in broilers
Source: J Anim Sci Biotechnol. 2025 Feb 24;16:28. doi: 10.1186/s40104-025-01163-3 (PMC11849324; doi:10.1186/s40104-025-01163-3)
Supplement: Supplementary file 1 — Additional file 1: Table S1. Composition of basal diet. [file 40104_2025_1163_MOESM1_ESM.docx]

**Supplementary materials**

**Table S1** Composition of basal diet (as fed)

| Items | Diet % |
| --- | --- |
| Corn | 65.80 |
| Soybean meal (46%) | 23.40 |
| Corn gluten meal | 5.00 |
| Soybean Oil | 2.10 |
| Limestone | 1.20 |
| Calcium hydrogen phosphate | 1.00 |
| NaCl | 0.1650 |
| Propionic acid antifungal agent ^d^ | 0.0518 |
| Premix of trace elements ^a^ | 0.1037 |
| Vitamin premix ^b^ | 0.0311 |
| L-Lysine | 0.3628 |
| DL Methionine | 0.1451 |
| L-Threonine | 0.0311 |
| Choline chloride | 0.1037 |
| Antioxidants ^e^ | 0.0104 |
| Heat-resistant phytase ^d^ | 0.0104 |
| AB Enzyme Complex ^c^ | 0.0031 |
| Sodium humate | 0.2073 |
| Mannanase (K302) ^d^ | 0.0104 |
| Sodium bicarbonate | 0.1555 |
| Zeolite ^d^ | 0.1161 |
| Total | 100 |
| Nutrient content |  |
| Metabolizable energy (kcal/kg) | 3050 |
| Crude protein (%) | 19.187 |
| Calcium (%) | 0.760 |
| Available phosphorus (%) | 0.487 |
| Lysine (%) | 1.103 |
| Methionine (%) | 0.411 |
| Methionine + Cystine (%) | 0.839 |
| Threonine (%) | 0.794 |
| Tryptophan (%) | 0.215 |

Note: ^a^ Trace element premix (mg/kg feed): Mn 100, Fe 80, Cu 8, Zn 75, Se 0.15, I 0.35. ^b^ Vitamin premix (provided per kilogram of feed): Vitamin A 12500 IU, Vitamin B1 2 mg, Vitamin B2 6 mg, Vitamin B12 0.025 mg, Vitamin D3 2500 IU, Vitamin E 30 IU, Vitamin K3 2.65 mg, pantothenic acid 12 mg, biotin 0.0325 mg, niacin 50 mg, folic acid 1.25 mg. ^c^ AB Enzyme Complex: Acid pro-tease, Neutral proteases, Xylanase, Cellulase, Glucoamylase. ^d^ Heat-resistant phytase, Man-nanase (K302) and Zeolite are provided by SUNHY Co (Wuhan, China), propionic acid antifun-gal agent is provided by KEMIN (China) Technology Co (Guangzhou, China). ^e^ A mixer of ethoxyquin (EQ), butylated hydroxytoluene (BHT) and butyl hydroxy anisd (BHA).
